# Regenerative Features of Adipose Tissue for Osteoarthritis Treatment in a Rabbit Model: Enzymatic Digestion Versus Mechanical Disruption

**DOI:** 10.3390/ijms20112636

**Published:** 2019-05-29

**Authors:** Giovanna Desando, Isabella Bartolotti, Lucia Martini, Gianluca Giavaresi, Nicolò Nicoli Aldini, Milena Fini, Alice Roffi, Francesco Perdisa, Giuseppe Filardo, Elizaveta Kon, Brunella Grigolo

**Affiliations:** 1 Laboratorio RAMSES, IRCCS Istituto Ortopedico Rizzoli, 40136 Bologna, Italy; giovanna.desando@ior.it (G.D.); isabella.bartolotti@ior.it (I.B.); brunella.grigolo@ior.it (B.G.); 2 Laboratory of Preclinical and Surgical Studies, IRCCS Istituto Ortopedico Rizzoli, 40136 Bologna, Italy; lucia.martini@ior.it (L.M.); gianluca.giavaresi@ior.it (G.G.); nicolo.nicolialdini@ior.it (N.N.A.); milena.fini@ior.it (M.F.); 3 Applied and Translational Research Center, IRCCS Istituto Ortopedico Rizzoli, 40136 Bologna, Italy; giuseppe.filardo@ior.it; 4 Hip and knee replacement Department, IRCCS Istituto Ortopedico Rizzoli, 40136 Bologna, Italy; francesco.perdisa@ior.it; 5 Humanitas University Department of Biomedical Sciences, Humanitas Clinical and Research Center, 20121 Milan, Italy; Elizaveta.kon@humanitas.it

**Keywords:** osteoarthritis, expanded adipose-derived stromal cells, adipose niche, local biodistribution, cartilage, synovial membrane, meniscus, CD-163 macrophages

## Abstract

Evaluating cell migration after cell-based treatment is important for several disorders, including osteoarthritis (OA), as it might influence the clinical outcome. This research explores migrating expanded-adipose stromal cells (ASCs) and adipose niches after enzymatic and mechanical processes. Bilateral anterior cruciate ligament transection induced a mild grade of OA at eight weeks in adult male New Zealand rabbits. ASCs, enzymatic stromal vascular fraction (SVF), and micro fragmented adipose tissue (MFAT) were intra-articularly injected in the knee joint. Assessments of cell viability and expression of specific markers, including CD-163 wound-healing macrophages, were done. Cell migration was explored through labelling with PKH26 dye at 7 and 30 days alongside co-localization analyses for CD-146. All cells showed good viability and high percentages of CD-90 and CD-146. CD-163 was significantly higher in MFAT compared to SVF. Distinct migratory potential and time-dependent effects were observed among cell-based treatments. At day 7, both ASCs and SVF migrated towards synovium, whereas for MFAT versus cartilage, a different migration pattern was noticed at day 30. The long-term distinct cell migration of ASCs, SVF, and MFAT open interesting clinical insights on their potential use for OA treatment. Moreover, the highest expression of CD-163 in MFAT, rather than SVF, might have an important role in directly mediating cartilage tissue repair responses.

## 1. Introduction

Osteoarthritis (OA) is a major chronic illness currently impacting a large group of patients, with rising costs for the European healthcare system. The lack of long-term disease-modifying treatments worsens the economic OA burden. Therefore, the search for innovative opportunities is tackled by scientists to improve current therapeutic strategies [1]. Among different approaches, the use of cell-based therapies has garnered considerable attention for promoting repair tissue responses due to cell differentiation and trophic activities [2]. Adipose-derived stromal cells (ASCs) represent good candidates, as they are available easily in a considerable amount through liposuction procedures. The repair potential of ASCs obtained a broad translational significance in a range of diseases, including OA [3,4]. Preclinical and clinical studies, based on the use of ASCs, reported encouraging results in modulating biological tissue responses and showed satisfactory results in OA patients. This effect is because of the higher immunomodulatory capacity of ASCs than bone marrow-derived mesenchymal stromal cells [5,6,7,8]. Indeed, ASCs can mediate, through trophic activities, the release of anabolic, anti-fibrotic, and anti-apoptotic growth factors within the OA microenvironment, contributing to reducing inflammatory and fibrosis responses [8,9,10].

Recently, the entire adipose niche has gained great attention because it is a reservoir of a heterogeneous cellular population, including progenitor cells, pericytes, endothelial cells, fibroblasts, pre-adipocytes, monocytes, macrophages, as well as an extracellular matrix (ECM) [11,12]. The biological significance in using this niche arises from the idea that the cross talk among progenitors and accessorise cells promote several repair and remodelling processes. The presence of immune cells and macrophages within the adipose niche can play a crucial role in mediating the clinical outcome. Macrophages can display classical inflammatory M1 [13,14] and, alternatively, wound-healing M2 [15,16] phenotypes that allow the participation of both pathological and physiological activities [9]. First applications of the adipose niche as an adipose stromal vascular fraction (SVF) were possible through the use of specific enzymatic treatments, including collagenase and trypsin [17]. Several studies confirmed the clinical value of autologous adipose SVF alone or in combination with platelet-rich plasma (PRP) or hyaluronic acid (HA) for different orthopaedic applications, including OA [18,19]. Possible concerns about enzymatic treatment, cell manipulation, pathogen contamination, and other regulations moved scientists to search for new alternatives to obtain an intact adipose niche [20,21]. To this end, scientists took advantage of ultrasounds, pressure, shear, and mechanical forces to process adipose tissue using various closed devices [22]. In particular, the Food and Drug Administration (FDA) defined mechanical effects among the minimal manipulations [23]. Besides EU legislation rules and safety considerations, the research focus aims to answer important biological questions to predict and assess the efficiency of potential therapeutic strategies. The impact of cell fate on repair mechanisms is being increasing accepted, because low and inefficient cell homing into injury sites can lead to a poor clinical outcome, thereby increasing the risk of failure [24,25]. Indeed, evaluating cell distribution and safety are among the main requirements for the use of advanced-therapy medicinal products [26,27]. Recently, our group has demonstrated the ASCs migration towards the synovial membrane and the meniscus following an intra-articular delivery in a preclinical animal model of OA, with various chondroprotective effects [8]. It remains necessary to clarify how the SVF and the micro fragmented adipose tissues (MFAT), after enzymatic and mechanical treatments, respectively, regulate cell homing in joint tissues in an OA setting. Therefore, the main aim of this study was to evaluate the migration pattern of expanded ASCs, SVF, and MFAT, delivered via an intra-articular (IA) route into the knee joint in a preclinical in vivo OA model.

## 2. Results

### 2.1. Expanded-ASCs, SVF, and MFAT Displayed Good Cell Viability and Expression of Progenitor Markers

**Figure 1:**
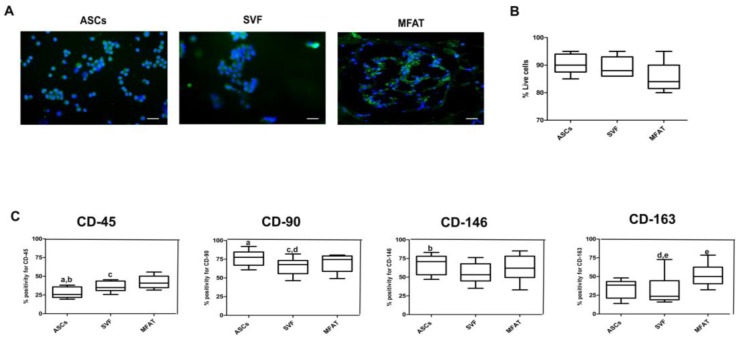
Viability and characterisation assessments of expanded-adipose stromal cells (ASC), stromal vascular fraction (SVF), and micro fragmented adipose tissue (MFAT). (**A**) Representative micrographs of Live/Dead analyses; blue staining: nuclei counterstaining; green staining: viable cells; red staining: dead cells; scale bar: 100 µm; (**B**) Bar graph reporting % of live cells for ASCs, SVF, and MFAT compounds as 95% confidence intervals (CI) of the mean ± standard deviation (SD). (**C**) Graphical representation of protein expression for CD-45, CD-90, CD-146, and CD-163 in ASCs, SVF and MFAT compounds detected by immunofluorescence analysis. Data are shown at 95% confidence intervals (CI) of the mean ± standard deviation (SD). Data were considered significant with *p* < 0.05: (a) % CD45 versus CD-90 in the ASC group; (b) % CD-45 versus CD-146 in the ASC group; (c) % CD-45 versus CD-90 in the MFAT group; (d) % CD-90 versus CD-163 in the SVF group; (e) % CD-163 in the SVF versus % CD-163 in the MFAT group.

ASCs displayed a spindle-shaped morphology, whereas both MFAT and SVF showed a mixed population of polygonal and fibroblastic cells, some containing lipid droplets in their cytoplasm. SVF and MFAT displayed cell population doublings comparable to ASCs. A Live/ Dead assay showed that all cell-based treatments displayed good percentages of cellular viability (Figure 1A). The mean SVF yield following enzymatic digestion ranged from 7 to 10 × 10^5^ nucleated cells per gram of adipose tissue. Due to the physical consistency of MFAT, it was not possible to perform a direct cell count. Haematoxylin and Eosin staining gave evidence of a mesh network containing blood vessels, pre-adipocytes with lipid droplets, and monocytes in the MFAT compound. Phenotypical analyses on ASCs, SVF, and MFAT gave evidence of strong positivity for the mesenchymal markers, CD-146 and CD-90, and a low percentage for the hematopoietic marker, CD-45. In particular, ASCs displayed higher CD90 and CD-146 protein expression compared to the CD-45 marker (*p* < 0.01 and *p* < 0.05, respectively). SVF showed a higher expression for CD-90 compared to CD-163 and CD-45 markers (*p* < 0.05). A higher level of CD-90 was evident in MFAT group compared to the CD-45 marker (*p* < 0.05). In general, MFAT displayed a higher CD-163 protein expression when compared to SVF product (*p* < 0.05). ASC treatment displayed the lowest CD-45 protein level when compared to the SVF group (*p* < 0.05) (Figure 1B).

### 2.2. ASCs, SVF, and MFAT Treatments Displayed a Distinctive Migration Pattern in the Synovial Membrane at 7 and 30 Days

**Figure 2:**
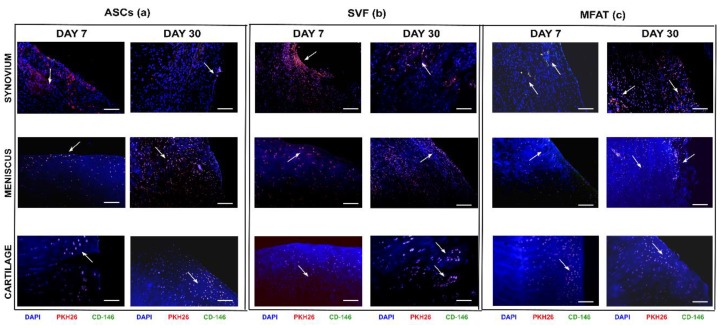
Representative micrographs of local biodistribution analyses at 7 and 30 days after intra-articular delivery of ASCs, SVF, and MFAT in the synovial membrane, the meniscus and the cartilage in an osteoarthritis (OA) rabbit model. (**a**) ASCs, (**b**) SVF, and (**c**) MFAT. Scale bar: 100 µm. Blue staining: nuclei counterstaining (dihydrochloride hydrate (DAPI) channel); red staining: PKH26 cell-labelling (Tetramethyl Rhodamine Iso-Thiocyanate (TRITC) channel); green staining: CD-146^+^ cells (with fluorescein isothiocyanate (FITC) channel); yellow staining: co-localization of PKH26^+^/CD-146^+^ cells. Black arrows: indications of some co-localized areas.

**Figure 3:**
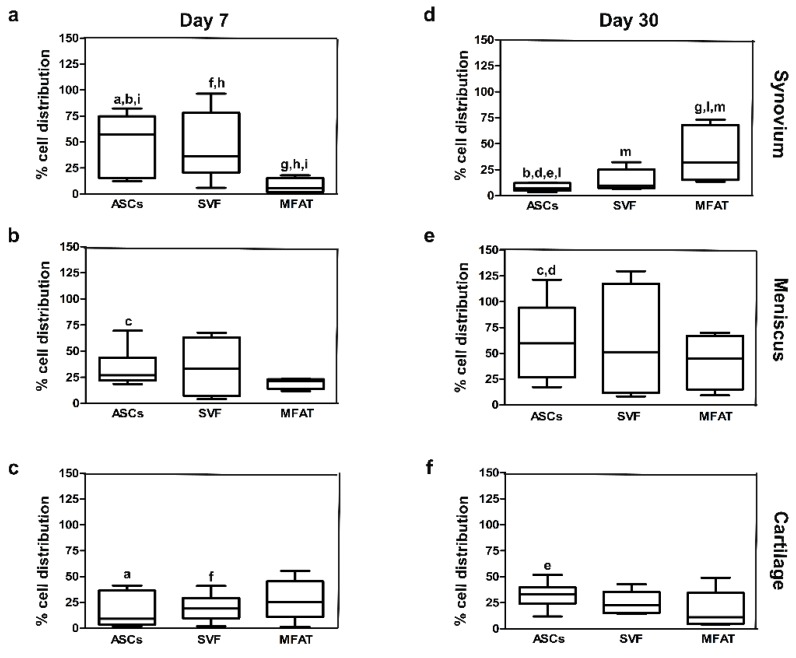
Graphical representation of local biodistribution analyses at day 7 (**a**–**c**) and day 30 (**d**–**f**) after intra-articular delivery of ASCs, SVF, and MFAT in the synovial membrane, the meniscus and the cartilage in a preclinical model. Data are shown at 95% confidence intervals (CI) of the mean ± standard deviation (SD). Data were considered significant with *p* < 0.05. (a) ASCs in synovium versus ASCs in cartilage at day 7; (b) ASCs at day 7 versus day 30 in synovium; (c) ASCs in cartilage at day 7 versus day 30 in the meniscus; (d) ASCs in synovium versus ASCs in the meniscus at day 30; (e) ASCs in synovium versus ASCs in cartilage at day 30; (f) SVF in synovium versus SVF in cartilage at day 7; (g) MFAT at day 7 versus day 30 in synovium; (h) SVF versus MFAT in synovium at day 7; (i) ASCs versus MFAT in synovium at day 7; (l) ASCs versus MFAT in synovium at day 30; (m) SVF versus MFAT in synovium at day 30.

The IA delivery of the different treatments into the OA knee joint did not have severe side effects at both experimental time points. Quantification through image analysis allowed the count of cell distribution for each joint tissue. ASC, SVF, and MFAT treatments showed different migration patterns in joint tissues at 7 and 30 days (Figure 2 and Figure 3). At day 7, ASCs showed higher tropism towards the synovial membrane compared to cartilage (*p* < 0.0005) (Figure 3a,c), whereas its percentage dropped at day 30 in the synovial membrane (*p* < 0.0005) (Figure 3d). The meniscus showed a moderate number of ASCs (30%) at day 7, and its expression resulted highly increased at day 30 (*p* < 0.01) (Figure 3b,e). At day 30, ASCs migrated especially in the meniscus, when compared to the synovial membrane (*p* < 0.01), and cartilage (*p* < 0.01) (Figure 3d–f).

Similarly to ASCs, SVF displayed higher tropism towards the synovial membrane and the meniscus, whereas the cartilage showed the lowest cell percentages on day 7. In particular, a higher rate of migrating cells from SVF was detected in the synovial membrane when compared to cartilage (*p* < 0.01) (Figure 3a,c). At day 30, the lowest number of SVFs was detected in the synovial membrane, whereas a higher percentage was detected in the meniscus and cartilage but with no statistical evidence (Figure 3d–f). As for the MFAT, most of the cells migrated towards the cartilage on day 7, with a reduction at long-term follow-up (Figure 3 c,f). At day 7, MFAT displayed the lowest tropism for the synovial membrane; however, a noticeable increase was detected at day 30 (*p* < 0.01) (Figure 3a,d). Expanded-ASCs, SVF, and MFAT showed different migration patterns in the synovial membrane at both time points. At day 7, MFAT displayed a lower cell migration in the synovial membrane compared to SVF (*p* < 0.01) and ASCs (*p* < 0.0005) groups. At day 30, MFAT showed, instead, a higher migration to the synovial membrane when compared to SVF (*p* < 0.05) and ASCs (*p* < 0.05) (Figure 3). Cartilage and meniscus tissues did not show significant differences among cell-based treatments at both time points (Figure 3).

### 2.3. Expanded-ASCs, SVF, and MFAT Treatments Displayed Different Percentages of CD-146^+^ Cells in the Cartilage and the Synovial Membrane

**Figure 4:**
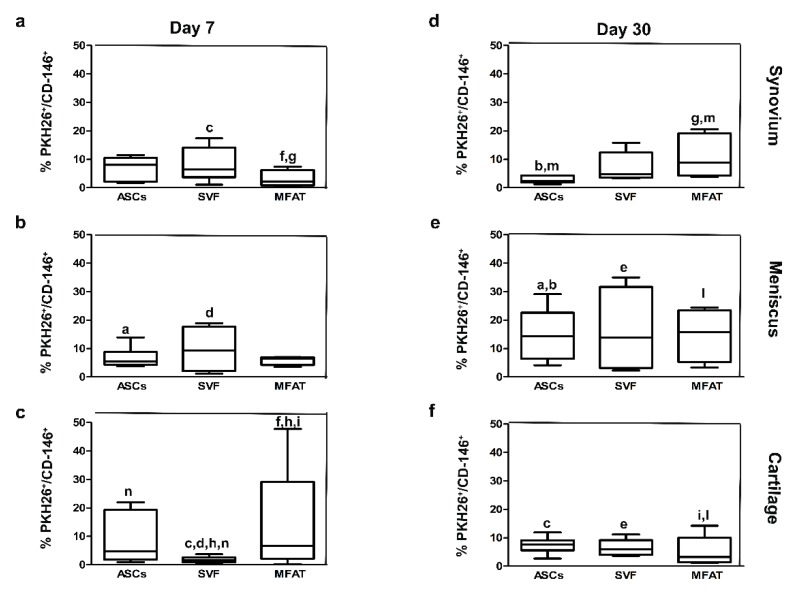
Graphical representation of the co-localized expression of progenitor cells and PKH26-labelled cells at day 7 (**a**–**c**) and day 30 (**d**–**f**) after intra-articular delivery of ASCs, SVF, and MFAT in the synovial membrane, the meniscus, and the cartilage. Data are shown at 95% confidence intervals (CI) of the mean ± standard deviation (SD). Data were considered significant with *p* < 0.05. (**a**) % PKH26 ^+^ /CD-146^+^ in the ASCs at day 7 versus ASCs at day 30 in the meniscus; (**b**) % PKH26^+^/CD-146^+^ in the ASCs in the synovium versus the ASCs in the meniscus at day 30; (**c**) % PKH26^+^/CD-146^+^ in SVF in the synovium versus SVF in cartilage at day 7; (**d**) % PKH26^+^/CD-146^+^ in SVF in meniscus versus SVF in cartilage at day 7; (**e**) % PKH26^+^/CD-146^+^ in the meniscus versus SVF in cartilage at day 30; (**f**) % PKH26^+^/CD-146^+^ in the MFAT in the synovium versus MFAT in cartilage at day 7; (g) % PKH26^+^/CD-146^+^ in MFAT at day 7 versus MFAT at day 30 in the synovium; (**h**) % PKH26^+^/CD-146^+^ in the SVF versus MFAT at day 7 in the cartilage; (**i**) % PKH26^+^/CD-146^+^ in MFAT at day 7 versus day 30 in cartilage; (**l**) % PKH26^+^/CD-146^+^ in MFAT in meniscus versus MFAT in cartilage at day 30; (m) % PKH26^+^/CD-146^+^ in ASCs versus MFAT in the synovial membrane at day 30. (n) % PKH26^+^/CD-146^+^ in ASCs versus SVF in the cartilage at day 7.

To explore the repair features of ASCs, SVF, and MFAT, co-localization analyses between CD-146, a reliable marker of pericytes, and PKH26-labelled cells were carried out (Figure 4a–f). ASCs showed similar levels of CD-146 in all joint tissues at day 7 (Figure 4a–c). The percentage of CD-146 progenitors in the ASC group increased in the meniscus from 7 to 30 days (*p* < 0.05) (Figure 4a,d). Herein, the percentages of CD-146^+^ cells were higher in the meniscus compared to the synovial membrane (*p* < 0.01) and cartilage (*p* < 0.05) at day 30 (Figure 4d–f). As for SVF, the lowest percentage of CD-146 progenitors was found in the cartilage on day 7, with a slight increase at day 30 (Figure 4c,f). At day 7, the SVF compound displayed higher levels of CD-146 progenitors near the synovial membrane and meniscus compared to cartilage (*p* < 0.05) (Figure 4a–c). The expression of CD-146^+^ cells was higher in the meniscus compared to the cartilage (*p* < 0.01) in the SVF group at day 30 (Figure 4e,f). MFAT showed a lower percentage of CD-146^+^ cells in the synovial membrane when compared to the cartilage at day 7 (*p* = 0.005) (Figure 4a,c). In particular, the presence of CD-146+ cells in the MFAT group showed a time-dependent effect in the synovial membrane, with an increased expression at day 30 (*p* < 0.05) (Figure 4a,d). At day 7, the highest expression of CD-146 was detected in the cartilage when compared to SVF (*p* < 0.0005) (Figure 4b,c), whereas its expression decreased from days 7 to 30 in the cartilage (*p* < 0.05) (Figure 4c,f). At day 30, the MFAT group showed the highest protein expression for the CD-146 marker in the meniscus compared to the cartilage (*p* < 0.05) (Figure 4e,f). Expanded-ASCs, SVF, and MFAT showed a significant amount of CD-146^+^ progenitor cells only in the cartilage and in the synovial membrane. Differences among tissues for each experimental group, and the experimental groups for each joint tissue, were analyzed. As for the cartilage, the percentage of CD-146 ^+^ /PKH26^+^ cells was higher in MFAT compared to the ASC group on day 7. Conversely, SVF displayed a lower amount of CD-146^+^/PKH26^+^ cells than the ASC group at day 7 (*p* < 0.01) (Figure 4). As for the synovial membrane, MFAT showed a greater number of CD-146^+^/PKH26^+^ cells than the ASC treatment (*p* < 0.05) at day 30 (Figure 4).

### 2.4. Expanded-ASCs, SVF, and MFAT Treatments Showed Different Histological Findings in the Cartilage and Synovial Membrane at 7 and 30 Days

**Figure 5:**
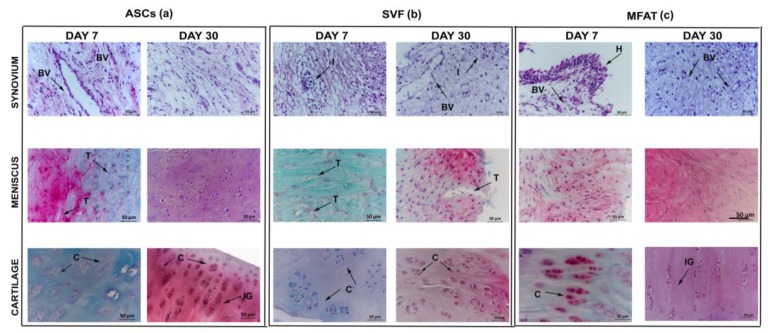
Representative micrographs of histological assessment of ASCs (**a**), SVF (**b**), and MFAT (**c**) in the synovial membrane; the meniscus and the cartilage were stained with Hematoxylin/Eosin and Safranin-O/Fast Green, respectively, at 7 and 30 days. Scale bar: 50 µm. Black arrows: indications of some histological details in tissue specimens. BV: blood vessels; I: inflammatory processes; H: hyperplastic and hypertrophic processes in the synovial membrane; T: tear presence in meniscus C: cell clones within the extracellular matrix in cartilage; F: fibrillation processes; IG: isogenic groups.

**Figure 6:**
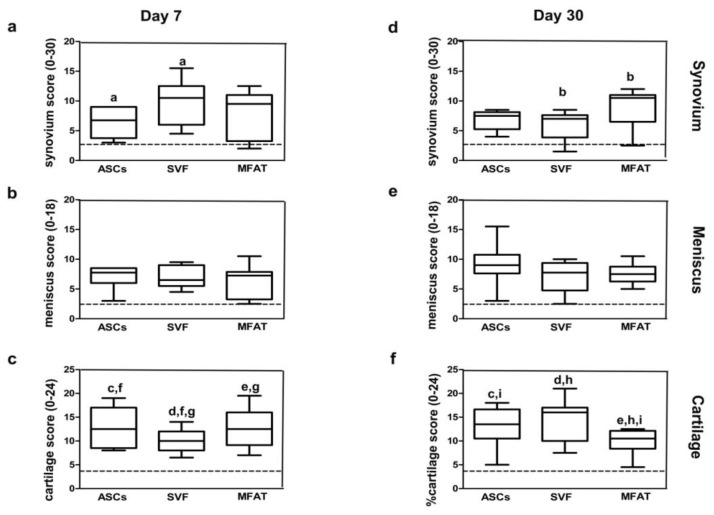
Graphical representation of synovium (Laverty’s score), meniscus (Modified Pauli’s score), and cartilage (Laverty’ score) at day 7 (**a**–**c**) and day 30 (**d**–**f**) after intra-articular delivery of ASCs, SVF, and MFAT in the synovial membrane, the meniscus and the cartilage. Data are shown at 95% confidence intervals (CI) of the mean ± standard deviation (SD). Data were considered significant with *p* < 0.05. Black dashed lines report the values of the sham group from a previous study. (**a**) Synovium score of the ASC group versus the SVF group at day 7; (**b**) synovium score of the SVF group versus the MFAT group at day 30; (**c**) cartilage score of the ASC group at day 7 versus ASCs at day 30; (d) cartilage score of the SVF group at day 7 versus SVF at day 30; (**e**) cartilage score of MFAT at day 7 versus MFAT at day 30; (**f**) cartilage score of ASCs versus SVF at day 7; (**g**) cartilage score of SVF versus MFAT at day 7; (**h**) cartilage score of SVF versus MFAT at day 30; (**i**) cartilage score of ASCs versus MFAT at day 30.

Cartilage, synovial membrane, and meniscus showed various histological features after ASCs, SVF and MFAT treatments at 7 and 30 days (Figure 5a–c). The ASC, SVF, and MFAT groups displayed mild hypertrophy and hyperplasia in the lining layer of the synovial membrane. At day 7, the ASC and SVF groups presented a subintimal layer made up of a low number of inflammatory cells and the presence of several blood vessels. MFAT treatment showed, instead, moderate pro-inflammatory cellular infiltrates around the peri-vascular regions and a high amount of blood vessels. Some degenerative features in the synovial stroma and a higher proliferation of fibroblasts were found mainly in the MFAT than in the ASC and SVF groups at day 7 (*p* < 0.01) (data not shown). Expanded-ASCs, SVF, and MFAT showed statistically different biological features in the synovial membrane at both time points. At day 7, SVF showed a higher score than the ASC treatment (*p* < 0.05), whereas MFAT did not show any statistical evidence. At day 30, MFAT displayed a higher histological score than SVF treatment (*p* < 0.01) reflecting the worst histological scenario (Figure 6).

All groups displayed similar histological features of the meniscus, although ASCs showed the best tissue architecture at 7 rather than 30 days (*p* < 0.0005) (data not shown). The SVF group presented some horizontal tears in the superficial and mid meniscus regions with glycosaminoglycans in the inner body of tissue. No significant changes for matrix organisation and Safranin O staining were evident among all cell-based treatments. About the cellular arrangement, ASC treatment showed similar results at both time points, whereas both SVF and MFAT displayed some changes from 7 to 30 days (*p* < 0.01) (data not shown). At day 30, the ASC group displayed cells with abundant cytoplasm, whereas the SVF and MFAT groups presented round and elongated cells, respectively. In general, the expanded-ASCs, SVF, and MFAT did not show histological differences at both time points (Figure 6).

As for cartilage, the ASC group showed an irregular tissue surface with the presence of fissures at day 7, as well as an extracellular matrix with a low proteoglycan content, and several cell clusters in the deep cartilagineous layer. At day 30, this treatment determined an increased proteoglycan content, a good cell organisation with round cells, and isogenous groups in the superficial and mid tissue layers, respectively (Figure 5a). The ASC group did not show any significant evidence between the two experimental times. The SVF group displayed tissue delamination, proteoglycan depletion, altered cell arrangement, and the presence of Safranin O negative cell clones, mainly near the deep cartilage zone. At day 30, SVF treatment contributed to improving tissue quality but with no statistical evidence. The extracellular matrix of the SVF group presented several areas of the Safranin O positive cell clones and an increased presence of proteoglycan within the extracellular matrix. The MFAT group showed significant cartilage histological improvements from 7 to 30 days. In particular, it displayed an increase of proteoglycan content (*p* < 0.05) and a reduction of cell clones and fissuration processes (*p* < 0.0005) (Figure 5). Expanded-ASCs, SVF, and MFAT gave evidence of several differences in cartilage score at both time points. At day 7, SVF displayed a lower score than the MFAT (*p* < 0.01) and ASC (*p* < 0.01) treatments. At day 30, MFAT showed a higher score than SVF (*p* < 0.0005) and ASCs (*p* < 0.01). Both SVF and MFAT groups displayed time-dependent effects for the cartilage. In particular, SVF showed the worst histological features from 7 to 30 days (*p* < 0.0005), whereas the ASC group showed a better histological scenario (*p* = 0.004) (Figure 6).

## 3. Discussion

Intra-articular injection of ASCs is considered one of the main delivery routes for OA treatment by allowing, with minimal invasiveness, a direct cell release into the injury site and the synthesis of active bio-molecules to foster tissue repair [28,29]. Possible concerns related to the risk of infection and genetic instability encouraged scientists to search for new alternatives for isolating SVF from the adipose tissue [22,23,30,31]. The great emphasis on the use of the adipose niche is due to its micro-architecture, being a rich source of progenitor and immune cells [11,12], and to its natural features [32,33]. The biological significance of recruiting progenitor cells in the injured sites contributes to promoting tissue repair. So far, expanded-MSCs engraft to injured sites to re-establish the impaired tissue homeostasis [8,27,34]. Although cells can attach to and penetrate joint tissues, they could undergo clearance from the joint space without guaranteeing long-term efficacy, thus limiting clinical success. This issue poses an urgent biological need, and evaluating cell migration can represent a valuable tool to predict the effectiveness of potential therapies and, thus, facilitate clinical decision making. Several studies analysed ASC behaviour in various preclinical models of OA. However, there are not many studies on SVF and MFAT therapies [8,19,35,36,37]. Therefore, the main objective of this study is to evaluate the migratory profile of the adipose niche from standard SVF and MFAT and expanded-ASCs at short and long-term follow-ups.

Moreover, this study provides preliminary insights on the histopathological features of OA joint tissues after cell treatments up to a one-month follow-up. Methods for isolating ASCs, SVF, and MFAT rely on enzymatic and mechanical techniques. Based on our previous study, a selected concentration of 2 × 10^6^ ASCs [8] and the processing of 10 mL of adipose tissue for preparing standard SVF and MFAT were carried out. SVF showed a cell yield ranging from 7 × 10^5^ to 10 × 10^5^ nucleated cells per gram of adipose tissue. However, no cell count was possible for the MFAT containing a heterogeneous cell population entrapped in an extracellular matrix. Like the ASCs and SVF, MFAT displayed a great positivity for CD-90 and CD-146 and a low percentage of CD-45^+^ cells [38,39]. Further, MFAT showed a higher protein expression for CD-163 marker than SVF, suggesting the mechanical process did not reduce the M2 macrophage subset [15,16]. Extensive *in vitro* studies on MFAT demonstrated the presence of an adipose niche rich of pericytes, showing the potential to differentiate towards osteogenic, chondrogenic, and adipogenic lineage and a low expression of basal inflammatory factors [39,40,41].

First analyses evaluated the cellular viability of expanded-ASCs, SVF, and MFAT because of the possible concerns arising from the use of enzymatic and mechanical processes [42]. Here, all treatments showed a high percentage of cell viability [39]. To explore cell migration, we set up a rabbit model of OA and performed in vitro cell labelling with PKH26, a lipophilic membrane binding dye, exploring cell distribution for long-term follow-up [43]. The expanded-ASC, SVF, and MFAT groups displayed distinctive migratory patterns only in the synovial membrane. In particular, ASCs and SVF displayed a high tropism for the synovial membrane, likewise for MFAT in cartilage at day 7. An opposite trend was noticed at day 30. It is possible to propose different hypotheses on the distinct migratory potential of ASCs, SVF, and MFAT. First, the physical features of cell preparations can influence their migration. ASCs and SVF are cell suspensions, while MFAT contains a variety of cells within a mesh of a collagen fibre network. Likely, the structure of MFAT could allow the long-term survival of cells in hypoxic tissues like cartilage by avoiding the degradation by enzymes, thus ensuring a gradual release of cytokines over time [44]. To date, the physiological hypoxic conditions of the cartilage are enhanced in OA due to the oxygen consumption by the synovial membrane and a reduction of O_2_ tension in the synovial fluid [45]. Although SVF and MFAT display biological similarities [42], their different migrations are likely dependent on the methods used for isolating stromal vascular fraction. To this end, we found that expanded-ASCs and SVF, both isolated with enzymatic techniques, showed lower levels of the CD-163 marker when compared to MFAT. The different migration pattern of MFAT at long-term follow-up could instead result from the physiological degradation of its collagen network. Indeed, the long-lasting presence of labelled cells from the ASC, SVF, and MFAT groups, and the positivity for CD-146 marker, could have clinical significance, ensuring a cellular reservoir based on the body’s demands.

In general, all treatments presented several repair responses in joint tissues. Similar to the ASC treatment, MFAT already showed cartilage repair at one month. MFAT displayed a prolonged secretory activity through the release of a granulocyte-colony stimulating factor (G-CSF) and hepatocyte growth factor (HGF) involved in cartilage repair [46]. However, MFAT displayed lower repair processes in the synovial membrane than ASC and SVF treatments at one month. Recently, Paolella et al. reported the active role of MFAT in mediating the activity of synovial macrophages through a decrease of CCL2/MCP1 and CCL3/MIP1α in a preclinical *in vitro* study [41]. Moreover, Nava S et al. demonstrated that MFAT releases mediators with more long-lasting anti-inflammatory properties than mesenchymal stromal cells under serum-free cell culture conditions [46]. Therefore, further studies are necessary to unravel the interplay between MFAT and the synovial membrane, as this anatomical site has an essential role in mediating OA changes and symptoms [47].

Findings from this study propose some preliminary explanations about the mechanisms of action of expanded-ASCs, SVF, and MFAT. While several authors have proven the effectiveness of ASCs in osteoarticular diseases and OA through the reduction of hypertrophy, inflammation, and dedifferentiation of chondrocytes [8,48,49], little is known about SVF and, especially, MFAT. Preclinical and clinical studies on SVF demonstrated its chondroprotective, immunomodulatory, and angiogenic properties [18,23,43], suggesting its potential use for the treatment of osteochondral diseases. Recently, several authors reported some benefits in the use of MFAT by using a preclinical OA animal model and a prospective, non-randomized, interventional, single-centre, clinical trial for OA patients focused on the evaluation of proteoglycan [50,51]. Moreover, Jannelli E and Fontana A. suggested the use of MFAT to treat delamination and first and second degree chondral lesions [52].

In general, we can speculate that the synovial membrane mainly mediates the wound-healing activity of ASCs and SVF by switching- off inflammatory signals through the activation of M2 macrophages. To this end, Manferdini et al. demonstrated that ASCs reduce inflammation through the tcyclooxygenase 2 (COX-2)/prostaglandin E2 (PGE2) pathway by switching M1 inflammatory macrophages into an M2-like phenotype [48,53] in the synovial membrane. Similarly, Bowles AC et al. proved in an experimental autoimmune encephalomyelitis (EAE) model that both ASCs and SVF promote regulatory T cells and M2 wound-healing macrophages, thereby reducing neuroinflammation in the central nervous system [54]. As for MFAT, we speculate that this treatment can exert anti-inflammatory and repair activities directly on cartilage tissue due to its trophic activity and the wound-healing activity of CD-163^+^ macrophages contained in its niche. Further, G-CSF could contribute to promoting repair processes in OA tissues by inhibiting fibrosis, as some authors have recently demonstrated an anti-fibrotic response in a model of pulmonary fibrosis with the activation of the AKT signalling pathway [55]. On the other hand, HGF could promote an anti-inflammatory response through the inhibition of TNF-α [55], typically involved in inflammation in the OA context. This study has some limitations, including the lack of a sham-operated control for cell labelling, a high number of markers for cell characterization, and histological assessments at long-term follow-ups to elucidate the role of the adipose niche in tissue repair.

Nevertheless, this study suggests a validated OA animal model, whereby expanded-ASCs, SVF, and MFAT displayed a long-term and distinctive cell migration in the synovial membrane, thus opening interesting clinical insights on their potential use for OA treatment. Moreover, the highest expression of CD-163 in MFAT rather than SVF might have an important biological significance in directly mediating cartilage tissue repair responses.

## 4. Materials and Methods

### 4.1. Experimental Design

The experimental protocol and surgical procedures for the animal study were approved by the Ethical Committee of the Rizzoli Orthopedic Institute and authorised by the Italian Ministry of Health (n° 862/2015-PR del 24/08/2015). Experimental research was carried out in compliance with Italian Law and according to EC Rules (Law by the Decree, n.26/2014 with the “Guide for the Care and Use of Laboratory Animals”. After pharmacological premedication with i.m. administration of a mixture of ketamine and xylazine, general anaesthesia was maintained with spontaneous breathing with O_2_/air and isoflurane 2%–3% with a facial mask. OA was surgically induced by bilateral anterior cruciate ligament transection (ACLT) in 18 skeletally mature male New Zealand rabbits (Harlan Laboratories, Inc., San Pietro di Natisone—Udine, Italy) (3 ± 0.5 Kg) through the use of standardised and validated surgical procedures [27,56]. All animals underwent standard antibiotics and analgesic administration. Animals were housed individually in a controlled environment (22 ± 1 °C, 55 ± 5% HR) with free access to food and water and were maintained for at least ten days before the beginning of the surgical procedures for acclimatisation. A total of 18 animals were divided into 3 experimental groups with bilateral treatments as follows: i) micro-fragmented adipose tissue (MFAT), using the Lipogems^®^ kit (*n* = 6, 12 joints); ii) stromal vascular fraction (SVF) (*n* = 6, 12 joints); and iii) 2 × 10^6^ expanded-ASCs (*n* = 6, 12 joints). Seven and thirty days were chosen as experimental times to monitor cell migration. A sham-operated group and an untreated group at eight weeks from ACLT (OA group) from a previous study [8] were used as experimental controls for histological evaluations to minimise the number of animals according to the principle of the 3 Rs (Replacement, Reduction and Refinement). At the time of euthanasia, osteochondral samples from femoral condyles and tibial plateau, menisci, synovial membrane, and ligaments were collected and processed, as already described in our previous work [8,27].

### 4.2. In Vitro Cell Processing: Cell Viability and Morphology

Adipose tissue was harvested under general anaesthesia from the inguinal area of 18 rabbits to isolate and process three cell preparations (expanded-ASCs, SVF, and MFAT), for the following intra-articular (IA) delivered into the knee joint. Ten milliliters of adipose tissue were processed in a closed system using Lipogems^®^ processing kit (Lipogems International SRL, Milan, Italy) to obtain MFAT. In brief, adipose tissue was placed inside the Lipogems device containing beads and fragmented by using mild mechanical forces and reduction filters to remove red blood cells and oil residues, without the addition of enzymes [42]. A small amount of MFAT was immediately fresh-frozen in liquid nitrogen and stored at −80 °C until their processing to perform morphological and viability assessments. The remaining portion of MFAT was plated into the culture medium to evaluate the release of progenitor cells from MFAT. As for SVF, 10 mL of adipose tissue were processed with 0.4 U/mL of collagenase NB4 standard grade (Serva Electrophoresis, GmbH, Heidelberg, Germany) for 30 min at 37 °C, and then the enzymatic activity was blocked with an α-MEM (Gibco, Carlsbad, CA, USA) medium supplemented with 15% fetal bovine serum (FBS) and 0.05 g/mL penicillin G (Gibco). Samples were centrifuged at 600 g for 10 min, and the pellet was resuspended with the culture medium and plated into culture flasks. As for the expanded-ASCs, the SVF fraction was initially plated at a density of 4000 cells/cm^2^, and then adherent cells were cultured and expanded for two weeks (density 2000 cells/cm^2^) in a complete α-MEM medium (Gibco) up to passage 2 at 37 °C, 5% CO_2_ in a humidified Heracell 150 i incubator (Thermo Fisher Scientific, Waltham, MA, USA). At 80% of confluence before passaging, cells were detached with a trypsin-EDTA solution 0.25% (PAA Laboratories, Linz, Austria). Cell viability was assessed via Live/Dead staining (Thermo Fisher Scientific, Waltham, MA, USA) for MFAT, SVF, and expanded-ASCs, according to manufacturers’ protocol, and evaluated with DS-Ri2 microscope by with fluorescein isothiocyanate (FITC) and Tetramethyl Rhodamine Iso-Thiocyanate (TRITC) channels to evaluate the number of live and dead cells in six microscopic fields (10×), respectively. Cell preparations were evaluated by immunofluorescence analyses for CD-45, CD-90, CD-146, and CD-163. Assessments were carried out on slides following the fixation with 4% PFA. Cell preparations were incubated firstly with 1% bovine serum albumin (BSA) (Sigma-Aldrich, St Louis, MO, USA) to avoid unspecific bindings and then with monoclonal antibodies against mouse monoclonal CD-45 (5 µg/mL; Origene, Bangalore, India), CD-90 (2.5 µg/mL; Thermo Fisher Scientific, Waltham, MA, USA), CD-146 (5 µg/mL; AbD Serotec, Bath, United Kingdom), and CD-163 (1µg/mL; Abcam, Cambridge, United Kingdom) diluted in Trizma buffered saline (TBS) for one hour at room temperature (RT). After three washes with TBS, slides were incubated with anti-mouse secondary antibodies conjugated with fluorescein isothiocyanate (FITC) (5 µg/mL; Dako, Santa Clara, CA, USA). After incubation, slides were washed and mounted in a polyvinyl alcohol mounting medium with 1,4-Diazabicyclo[222]octane (DABCO) (Sigma-Aldrich) containing one µg/mL 4,6-diamidino-2-phenylindole dihydrochloride hydrate (DAPI; Sigma-Aldrich). A DS-Ri2 microscope (Nikon, Tokyo, Japan), containing NIS-Elements Software (Nikon, Tokyo, Japan), allowed the evaluation of cell preparations. A hue Saturation Intensity (HIS) system was used for image analysis. After having defined pixels for the negative controls, we evaluated percentages of positivity for the different markers, setting the Hue range values to 150–255 and saturation and the intensity range values to 20–255.

### 4.3. Cell Labelling and Preparation for Local Biodistribution Studies

MFAT, SVF, and expanded-ASCs were labelled with a PKH26 Red Fluorescent Cell Linker (Sigma-Aldrich), a lipophilic fluorescent membrane dye, and evaluated at 7 and 30 days following their intra-articular delivery in OA rabbits. In brief, cells were incubated for 5 min at room temperature (RT) with two µM PKH26 re-suspended in the PKH diluent, an iso-osmotic, salt and a solvent-free vehicle supplied within the kit. After the incubation, cell preparations were firstly placed in an α-MEM medium to stop the reaction and then washed five times with a physiological solution (PS) to remove unbound dye. After having obtained the labeled-MFAT, SVF, and 2 × 10^6^ expanded-ASCs, each cell preparation was re-suspended in 300 µl PS, and intra-articularly injected into the joint capsule after OA onset.

### 4.4. In Vivo Local Biodistribution: Cell Localisation and CD-146 Assessment

All animals have reached experimental times without complications during the postoperative period. At the scheduled experimental times, animals were euthanised under deep general anaesthesia with intravenous administration of Tanax^®^, and joint tissues were harvested. Femoral condyles, tibial plateau, menisci, and synovial membrane were fixed in 10% neutral buffered formalin and decalcified in 4% hydrochloric acid and 5% formic acid (Sigma-Aldrich) when the bone component was present, to be finally processed and have paraffin embedded. Tissue sections (5 µm) were cut and counterstained with one µg/mL DAPI (Sigma-Aldrich) for nuclear staining. Three slides for each tissue at different depth were analysed using a DS-Ri2 microscope (Nikon, Tokyo, Japan).

Moreover, we analysed the presence of a cluster of differentiation (CD)-146 in the labelled cells of each anatomical site through immunofluorescence analysis. To this end, sections were deparaffinized and incubated with 1% BSA (Sigma-Aldrich) for 10 min at RT and then with a monoclonal antibody against mouse monoclonal CD-146 (0.5 µg/mL; AbD Serotec, Dusseldorf, Germany). After washing with TBS, tissues were incubated with an anti-mouse secondary antibody conjugated with FITC (5 µg/mL; Dako). Samples were then washed with TBS and mounted in a polyvinyl alcohol mounting medium with DABCO containing DAPI (Sigma-Aldrich). After the immunofluorescence analysis, we acquired whole tissue sections using DAPI, FITC, and TRITC filters to evaluate nuclei, CD-146 positivity, and PKH-26 labelled cells, respectively. Then, we merged DAPI, FITC, and TRICT triple filters to one image for each tissue, on which we carried out the image analysis considering co-localization signals from the labelled-cells (TRITC filter) and the CD-146 marker (FITC filter). Quantification was expressed as a percentage of the co-localization of FITC^+^/TRITC^+^ signals using a Hue Saturation Intensity (HIS) system with NIS-Elements Software (Nikon, Tokyo, Japan) through a DS-Ri2 microscope. We set the threshold range values of Hue at 170–255 and Saturation and Intensity at 40–255.

### 4.5. Histological Analyses of Tissue Explants

Femoral condyles, synovial membranes, and meniscus specimens were processed as previously described. After tissue processing, osteochondral and meniscus specimens were stained with Safranin-O/Fast Green (Sigma Aldrich), whereas synovial membranes were stained with Haematoxylin/Eosin (Sigma Aldrich) to monitor proteoglycan/collagen content and tissue architecture, respectively. Seven sagittal osteochondral, meniscus and synovium sections, spaced ten sections apart, were graded for OA severity using validated semi-quantitative Laverty’s scores [57,58] and a modified Pauli’s score [27,59], respectively. In brief, Laverty’s scores for cartilage and synovium have a scale from 0 to 24 and 0 to 30, respectively. Laverty’s score for cartilage considered the following histopathological features: safranin-O/Fast green staining, cartilage structure, chondrocyte density, and cluster formation. The Laverty score for synovium takes into account the following histological features: synoviocytes (proliferation, hypertrophy), the inflammatory state, and the synovial stroma (hyperplasia, the proliferation of blood vessels, the proliferation of fibroblasts, and cartilage/bone detritus). The modified Pauli’s score takes into account the following histopathological features: surface, cellularity, matrix organisation, and matrix staining, and has a scale from 0 to 18. For all the proposed scores, the lowest values reflect healthy tissues, whereas the highest value reflect scenarios of severe OA. All evaluations were performed with a DS-Ri2 microscope (Nikon, Tokyo, Japan); the samples were blinded to the researchers.

### 4.6. Statistical Analysis

Statistical analysis was carried out using the Statistical Package for the Social Sciences (SPSS Inc., Chicago, IL, USA) software version 15.0 (SPSS Inc.). Data are reported at 95% confidence intervals (CI) of the mean ± standard deviation (SD). Post-hoc comparisons with Mann Whitney test, evaluated by Montecarlo Method for small samples and Bonferroni correction for multiple comparisons, were carried out to assess phenotypical differences among ASCs, SVF, and MFAT. The Friedman Wilcoxon posthoc test with Bonferroni correction was used for paired comparisons to evaluate the phenotypical differences of each group at the different experimental times.

The general linear model (GLM) with Sidak correction for multiple comparisons was used, instead, to assess the influence of the kind of treatment and experimental time on cell biodistribution, CD-146 protein expression in labelled cells within the joint tissues, and cartilage, synovium and meniscus scores. Data were considered significant with *p* < 0.05.

## References

[1] Turkiewicz A., Petersson I.F., Bjork J., Hawker G., Dahlberg L.E., Lohmander L.S., Englund M. (2014). Current and future impact of osteoarthritis on health care: A population-based study with projections to year 2032. Osteoarthr. Cartil..

[2] Andrzejewska A., Lukomska B., Janowski M. (2019). Concise Review: Mesenchymal Stem Cells: From Roots to Boots. Stem Cells.

[3] Ra J.C., Kang S.K., Shin I.S., Park H.G., Joo S.A., Kim J.G., Kang B.C., Lee Y.S., Nakama K., Piao M. (2011). Stem cell treatment for patients with autoimmune disease by systemic infusion of culture-expanded autologous adipose tissue-derived mesenchymal stem cells. J. Transl. Med..

[4] Spasovski D., Spasovski V., Baščarević Z., Stojiljković M., Vreća M., Anđelković M., Pavlovic S. (2018). Intra-articular injection of autologous adipose-derived mesenchymal stem cells in the treatment of knee osteoarthritis. J. Gene Med..

[5] Yañez R., Lamana M.L., García-Castro J., Colmenero I., Ramírez M., Bueren J.A. (2006). Adipose tissue-derived mesenchymal stem cells have in vivo immunosuppressive properties applicable for the control of the graft-versus-host disease. Stem Cells.

[6] Pagani S., Borsari V., Veronesi F., Ferrari A., Cepollaro S., Torricelli P., Filardo G., Fini M. (2017). Increased chondrogenic potential of mesenchymal cells from adipose tissue versus bone marrow-derived cells in osteoarthritic in vitro models. J. Cell Physiol..

[7] Gimble J.M., Guilak F., Bunnell B.A. (2010). Clinical and preclinical translation of cell-based therapies using adipose tissue-derived cells. Stem Cell Res. Ther..

[8] Desando G., Cavallo C., Sartoni F., Martini L., Parrilli A., Veronesi F., Fini M., Giardino R., Facchini A., Grigolo B. (2013). Intra-articular delivery of adipose derived stromal cells attenuates osteoarthritis progression in an experimental rabbit model Intra-articular delivery of adipose derived stromal cells attenuates osteoarthritis progression in an experimental rabbit model. Arthritis. Res. Ther..

[9] Rehman J., Traktuev D., Li J., Merfeld-Clauss S., Temm-Grove C.J., Bovenkerk J.E., Pell C.L., Johnstone B.H., Considine R.V. (2004). Secretion of angiogenic and antiapoptotic factors by human adipose stromal cells. Circulation.

[10] Puissant B., Barreau C., Bourin P., Clavel C., Corre J., Bousquet C., Taureau C., Cousin B., Abbal M. (2005). Immunomodulatory effect of human adipose tissue-derived adult stem cells: Comparison with bone marrow mesenchymal stem cells. Br. J. Haematol..

[11] Lee Y.H., Petkova A.P., Granneman J.G. (2013). Identification of an Adipogenic Niche for Adipose Tissue Remodeling and Restoration. Cell Metab..

[12] Kaewsuwan S., Song S.Y., Kim J.H., Sung J.H. (2012). Mimicking the functional niche of adipose-derived stem cells for regenerative medicine. Expert Opin. Biol. Ther..

[13] Pollard J.W. (2009). Trophic macrophages in development and disease. Nat. Rev. Immunol..

[14] Chawla A., Nguyen D.K., Goh Y.P.S. (2012). Macrophage-Mediated Inflammation in Metabolic Disease. Nat. Rev. Immunol..

[15] Odegaard J.I., Chawla A. (2011). Alternative Macrophage Activation and Metabolism. Annu. Rev. Path..

[16] Sica A., Mantovani A. (2012). Macrophage plasticity and polarization: In vivo veritas. J. Clin. Investig..

[17] Bourin P., Bunnell B.A., Casteilla L., Dominici M., Katz A.J., March K.L., Red H., Rubin J.P., Yoshimura K., Gimble J.M. (2013). Stromal cells from the adipose tissue-derived stromal vascular fraction and culture expanded adipose tissue-derived stromal/ stem cells: A joint statement of the International Federation for Adipose Therapeutics (IFATS) and Science and the International Society for Cellular Therapy (ISCT). Cytotherapy.

[18] Pak J., Lee J.H., Park K.S., Park M., Kang L.W., Lee S.H. (2017). Current use of autologous adipose tissue-derived stromal vascular fraction cells for orthopaedic applications. J. Biomed. Sci..

[19] Pak J., Lee J.H., Pak N., Pak Y., Park K., Jeon J.H., Jeong B.C. (2018). Cartilage Regeneration in Humans with Adipose Tissue-Derived Stem Cells and Adipose Stromal Vascular Fraction Cells: Updated Status. Int. J. Mol. Sci..

[20] Aronowitz J.A., Lockhart R.A., Hakakian C.S. (2015). Mechanical versus enzymatic isolation of stromal vascular fraction cells from adipose tissue. Springerplus.

[21] Shah F.S., Wu X., Dietrich M., Rood J., Gimble J.M. (2013). A non-enzymatic method for isolating human adipose tissue-derived stromal stem cells. Cytotherapy.

[22] Oberbauer E., Steffenhagen C., Wurzer C., Gabriel C., Red H., Wolbank S. (2015). Enzymatic and non-enzymatic isolation systems for adipose tissue-derived cells: Current state of the art. Cell Regen..

[23] Bora P., Majumdar A.S. (2017). Adipose tissue-derived stromal vascular fraction in regenerative medicine: A brief review on biology and translation. Stem Cell Res. Ther..

[24] Sarkar D., Spencer J.A., Phillips J.A., Zhao W., Schafer S., Spelke D.P., Luke J., Mortensen L.J., Ruiz J.P., Vemula P.K. (2011). Engineered cell homing. Blood.

[25] Su P., Tian Y., Yang C., Ma X., Wang X., Pei J., Qian A. (2018). Mesenchymal Stem Cell Migration during Bone Formation and Bone Diseases Therapy. Int. J. Mol. Sci..

[26] Sensebé L., Fleury-Cappellesso S. (2013). Biodistribution of mesenchymal stem/stromal cells in a preclinical setting. Stem Cells Int..

[27] Desando G., Bartolotti I., Cavallo C., Schiavinato A., Secchieri C., Kon E., Filardo G., Paro M., Grigolo B. (2018). Short-Term Homing of Hyaluronan-Primed Cells: Therapeutic Implications for Osteoarthritis Treatment. Tissue Eng. Part C Methods..

[28] Schäffler A., Büchler C. (2007). Concise Review: Adipose Tissue-Derived Stromal Cells-Basic and Clinical Implications for Novel Cell-Based Therapies. Stem Cells.

[29] Perdisa F., Gostyńska N., Roffi A., Filardo G., Marcacci M., Kon E. (2015). Adipose-Derived Mesenchymal Stem Cells for the Treatment of Articular Cartilage: A Systematic Review on Preclinical and Clinical Evidence. Stem Cells Int..

[30] Dahl J.A., Duggal S., Coulston N., Millar D., Melki J., Shahdadfar A., Brinchmann J.E., Collas P. (2008). Genetic and epigenetic instability of human bone marrow mesenchymal stem cells expanded in autologous serum or fetal bovine serum. Int. J. Dev. Biol..

[31] Bedford P., Jy J., Collins L., Keizer S. (2018). Considering Cell Therapy product “Good Manufacturing practice” Status. Front. Med..

[32] Bateman M.E., Strong A.L., Gimble J.M., Bunnell B.A. (2018). Concise Review: Using Fat to Fight Disease: A Systematic Review of Nonhomologous Adipose-Derived Stromal/Stem Cell Therapies. Stem Cells.

[33] Russo A., Condello V., Madonna V., Guerriero M., Zorzi C. (2017). Autologous and micro-fragmented adipose tissue for the treatment of diffuse degenerative knee osteoarthritis. J. Exp. Orthop..

[34] Scanzello C.R. (2017). Chemokines and inflammation in osteoarthritis: Insights from patients and animal models. J. Orthop. Res..

[35] Cattaneo G., De Caro A., Napoli F., Chiapale D., Trada P., Camera A. (2018). Micro-fragmented adipose tissue injection associated with arthroscopic procedures in patients with symptomatic knee osteoarthritis. BMC Musculoskelet Disord..

[36] Arthurs J.R., Desmond C.R., TerKonda S.P., Shapiro S.A. (2018). Micro-fragmented adipose tissue for treatment of knee osteoarthritis with Baker’s cyst: A case study. BMJ Case Rep..

[37] Bright B., Bright R., Bright P., Limaye A. (2018). Ankylosing spondylitis, chronic fatigue and depression improved after stromal vascular fraction treatment for osteoarthritis: A case report. J. Med. Case Rep..

[38] Tremolada C., Colombo V., Ventura C. (2016). Adipose Tissue and Mesenchymal Stem Cells: State of the Art and Lipogems^®^ Technology Development. Curr. Stem Cell Rep..

[39] Bianchi F., Maioli M., Leonardi E., Olivi E., Pasquinelli G., Valente S., Mendez A.J., Ricordi C., Raffaini M., Tremolada C. (2013). A new nonenzymatic method and device to obtain a fat tissue derivative highly enriched in pericyte-like elements by mild mechanical forces from human lipoaspirates. Cell Transplant..

[40] Vezzani B., Shaw I., Lesme H., Yong L., Khan N., Tremolada C., Péault B. (2018). Higher pericyte content and secretory activity of micro fragmented human adipose tissue compared to enzymatically derived stromal vascular fraction. Stem Cells Transl. Med..

[41] Paolella F., Manferdini C., Gabusi E., Gambari L., Filardo G., Kon E., Mariani E., Lisignoli G. (2018). Effect of microfragmented adipose tissue on osteoarthritic synovial macrophage factors. J. Cell Physiol..

[42] Busser H., De Bruyn C., Urbain F., Najar M., Pieters K., Raicevic G., Meuleman N., Bron D., Lagneaux L. (2014). Isolation of adipose-derived stromal cells without enzymatic treatment: Expansion, phenotypical, and functional characterization. Stem Cells Dev..

[43] Shuai H., Shi C., Lan J., Chen D., Luo X. (2015). Double labelling of human umbilical cord mesenchymal stem cells with Gd-DTPA and PKH26 and the influence on biological characteristics of hUCMSCs. Int. J. Exp. Pathol..

[44] Swärd P., Wang Y., Hansson M., Lohmander L.S., Grodzinsky A.J., Struglics A. (2017). Coculture of bovine cartilage with synovium and fibrous joint capsule increases aggrecanase and matrix metalloproteinase activity. Arthritis. Res. Ther..

[45] Pfander D., Gelse K. (2007). Hypoxia and osteoarthritis: How chondrocytes survive hypoxic environments. Curr. Opin. Rheumatol..

[46] Nava S., Sordi V., Pascucci L., Tremolada C., Ciusani E., Zeira O., Cadei M., Soldati G., Pessina A., Parati E. (2019). Long-lasting anti-inflammatory activity of human micro-fragmented adipose tissue. Stem Cells Int..

[47] Scanzello C., Goldring S.R. (2012). The role of synovitis in osteoarthritis pathogenesis. Bone.

[48] Manferdini C., Maumus M., Gabusi E., Piacentini A., Filardo G., Peyrafitte J.A., Jorgensen C., Bourin P., Fleury-Cappellesso S., Facchini A. (2013). Adipose-derived mesenchymal stem cells exert anti-inflammatory effects on chondrocytes and synoviocytes from osteoarthritis patients through prostaglandin E2. Arthritis. Rheum..

[49] Maumus M., Manferdini C., Toupet K., Peyrafitte J.A., Ferreira R., Facchini A., Gabusi E., Bourin P., Jorgensen C., Lisignoli G. (2013). Adipose mesenchymal stem cells protect chondrocytes from degeneration associated with osteoarthritis. Stem Cell Res..

[50] Zeira O., Scaccia S., Pettinari L., Ghezzi E., Asiag N., Martinelli L., Zahirpour D., Dumas M.P., Konar M., Lupi D.M. (2018). Intra-articular administration of autologous micro fragmented adipose tissue in dogs with spontaneous osteoarthritis: Safety, feasibility and clinical outcomes. Stem Cells Int. Med..

[51] Hudetz D., Borić I., Rod E., Jeleč Ž., Radić A., Vrdoljak T., Skelin A., Lauc G., Trbojević-Akmačić I., Plečko M. (2017). The effects of Intra-articular Injection of Autologous Microfragmented Fat Tissue on Proteoglycan Synthesis in Patients with Knee Osteoarthritis. Genes.

[52] Jannelli E., Fontana A. (2017). Arthroscopic treatment of chondral defects in the hip: AMIC, MACI, micro fragmented adipose tissue transplantation (MATT)and other options. SICOT J..

[53] Manferdini C., Paolella F., Gabusi E., Gambari L., Piacentini A., Filardo G., Fleury-Cappellesso S., Barbero A., Murphy M., Lisignoli G. (2017). Adipose stromal cells mediated switching of the pro-inflammatory profile of M1-like macrophages is facilitated by PGE2: In vitro evaluation. Osteoarthr. Cartil..

[54] Bowles A.C., Wise R.M., Gerstein B.Y., Thomas R.C., Ogelman R., Febbo I., Bunnel B.A. (2017). Immunomodulatory Effects of Adipose Stromal Vascular Fraction Cells Promote Alternative Activation Macrophages to Repair Tissue Damage. Stem Cells.

[55] Zhao F., Liu W., Yue S., Yang L., Hua Q., Zhou Y., Cheng H., Luo Z., Tang S. (2019). Pretreatment with G-CSF Could Enhance the Antifibrotic Effect of BM-MSCs on Pulmonary Fibrosis. Stem Cells Int..

[56] Yoshioka M., Coutts R.D., Amiel D., Hacker S.A. (1996). Characterization of a model of osteoarthritis in the rabbit knee. Osteoarthr. Cartil..

[57] Laverty S., Girard C.A., Williams J.M., Hunziker E.B., Pritzker K.P. (2010). The OARSI histopathology initiative—Recommendations for histological assessments of osteoarthritis in the rabbit. Osteoarthr. Cartil..

[58] Chevrier A., Nelea M., Hurtig M.B., Hoemann C.D., Buschmann M.D. (2009). Meniscus structure in human, sheep, and rabbit for animal models of meniscus repair. J. Orthop. Res..

[59] Pauli C., Grogan S.P., Patil S., Otsuki S., Hasegawa A., Koziol J., Lotz M.K., D’Lima D.D. (2011). Macroscopic and histopathologic analysis of human knee menisci in aging and osteoarthritis. Osteoarthr. Cartil..

